# Contribution of germline *BRCA1 *and *BRCA2 *sequence alterations to breast cancer in Northern India

**DOI:** 10.1186/1471-2350-7-75

**Published:** 2006-10-04

**Authors:** Sunita Saxena, Anurupa Chakraborty, Mishi Kaushal, Sanjeev Kotwal, Dinesh Bhatanager, Ravindar S Mohil, Chintamani Chintamani, Anil K Aggarwal, Veena K Sharma, Prakash C Sharma, Gilbert Lenoir, David E Goldgar, Csilla I Szabo

**Affiliations:** 1 Institute of Pathology, Safdarjang Hospital Campus, New Delhi, India; 2 Unit of Genetic Epidemiology, International Agency for Research on Cancer, Lyon, France; 3 Safdarjang Hospital, New Delhi, India; 4 Department Of Pathology, L.L.R.M. Medical College, Meerut, India; 5 Guru Govind Singh Indraprastha University, Kashmiri Gate, Delhi, India; 6 Institut Gustave-Roissy, Villejuif, Paris, France; 7 Laboratory Medicine and Experimental Pathology, MayoClinic, Rochester, MN, USA

## Abstract

**Background:**

A large number of distinct mutations in the *BRCA1 *and *BRCA2 *genes have been reported worldwide, but little is known regarding the role of these inherited susceptibility genes in breast cancer risk among Indian women. We investigated the distribution and the nature of *BRCA1 *and *BRCA2 *germline mutations and polymorphisms in a cohort of 204 Indian breast cancer patients and 140 age-matched controls.

**Method:**

Cases were selected with regard to early onset disease (≤40 years) and family history of breast and ovarian cancer. Two hundred four breast cancer cases along with 140 age-matched controls were analyzed for mutations. All coding regions and exon-intron boundaries of the *BRCA1 *and *BRCA2 *genes were screened by heteroduplex analysis followed by direct sequencing of detected variants.

**Results:**

In total, 18 genetic alterations were identified. Three deleterious frame-shift mutations (185delAG in exon 2; 4184del4 and 3596del4 in exon 11) were identified in *BRCA1*, along with one missense mutation (K1667R), one 5'UTR alteration (22C>G), three intronic variants (IVS10-12delG, IVS13+2T>C, IVS7+38T>C) and one silent substitution (5154C>T). Similarly three pathogenic protein-truncating mutations (6376insAA in exon 11, 8576insC in exon19, and 9999delA in exon 27) along with one missense mutation (A2951T), four intronic alterations (IVS2+90T>A, IVS7+75A>T, IVS8+56C>T, IVS25+58insG) and one silent substitution (1593A>G) were identified in *BRCA2*. Four previously reported polymorphisms (K1183R, S1613G, and M1652I in *BRCA1*, and 7470A>G in *BRCA2*) were detected in both controls and breast cancer patients. Rare *BRCA1/2 *sequence alterations were observed in 15 out of 105 (14.2%) early-onset cases without family history and 11.7% (4/34) breast cancer cases with family history. Of these, six were pathogenic protein truncating mutations. In addition, several variants of uncertain clinical significance were identified. Among these are two missense variants, one alteration of a consensus splice donor sequence, and a variant that potentially disrupts translational initiation.

**Conclusion:**

*BRCA1 *and *BRCA2 *mutations appear to account for a lower proportion of breast cancer patients at increased risk of harboring such mutations in Northern India (6/204, 2.9%) than has been reported in other populations. However, given the limited extent of reported family history among these patients, the observed mutation frequency is not dissimilar from that reported in other cohorts of early onset breast cancer patients. Several of the identified mutations are unique and novel to Indian patients.

## Background

Breast cancer is the most prevalent malignancy and primary cause of cancer death in women worldwide. It accounts for 23% of all cancers among women, and is the second most common cancer overall when both sexes are considered together. Despite substantial differences in age-standardized incidence rates between developed and developing countries (age standardised rates per 100,000 women (ASR) ranging from 99.4 to 16.5 in North America and Central Africa, respectively), differential survival in developed versus developing countries diminishes the range observed in corresponding mortality rates (ASR of 19.2 in North America vs. 12.1 in Central Africa). In all, breast cancer accounts for 14.1% of female cancer deaths. Most alarmingly, incidence rates have continued to increase worldwide, with an overall annual increase of approximately 0.5% since 1990. However, changes in incidence rates are greatest in developing countries, attaining annual increases of 3–4%. Should these trends continue, it is estimated that 1.5 million new cases of breast cancer will be diagnosed in 2010 [1].

In India, an average of 80,000 women are diagnosed with carcinoma of the breast, and 40,000 women die of the disease every year [2]. Although it is currently the second most common cancer among Indian women (19%) after cervical cancer (30%), in the urban cancer registries of Delhi and Mumbai, breast cancer has rapidly overtaken cervical cancer in frequency. The highest cancer incidence rate recorded among women at the Delhi Cancer Registry is breast cancer (ASR 30.5). These data not only demonstrate the magnitude of the current health problem associated with breast cancer in the Indian population, but also indicate that socio-economic trends will lead to rapid increases in its contribution to the overall health care burden.

Interestingly, although overall incidence of breast cancer in Indian population is low compared to Western populations (ASR of 23.5 vs. 90.7), the incidence of early onset disease (< 40 yrs) does not show significant geographic variation (ASR range worldwide of 12–33) [3] suggesting that in the Indian population a greater proportion of breast cancer is due to early onset disease compared to Western populations. According to the National Cancer Registry Project [NCRP] based on cancer registries at six hospitals, the average age of patients was found to range from 44.2 years (Dibrugarh) to 49.6 years (Bangalore and Chennai) [4]. Similarly, the average age of breast cancer patients in various population-based registries throughout India has been reported to be 50–53 years [5] whereas amongst US white females it is 61.0 years [6] showing that in Indian women, disease occurs a decade earlier than in Western populations.

Several environmental risk factors that may contribute to or hasten the development of breast cancer have been identified, including mainly lifestyle and reproductive factors. These may account for the majority of observed trends and the variation in incidence rates between developed and developing countries. The factor with the strongest breast cancer risk association is a family history of breast and/or ovarian cancer, the associated risk being even higher for family history of early onset disease (≤ age 40). The majority of familial breast cancer cases present at an early age relative to sporadic cancer, and genetic factors are considered to play major role in their development. Hence, the earlier average age of breast cancer among Indian women is intriguing, suggestive of a significant genetic component in this population.

Genetic susceptibility to cancer is triggered in several ways, the best understood causal mechanism being due to inactivating germline mutations in tumor suppressor and DNA repair genes, which lead to an accumulation of mutations in oncogenes and cell-cycle checkpoints that are required for uncontrolled cell division. About 5–10% of breast and ovarian cancer occurs as a result of highly penetrant germline mutations. Two major breast cancer susceptibility genes are *BRCA1 *(MIM 113705, Genbank accession no. U14680) and *BRCA2 *(MIM 600185, Genbank accession no. U43746), located on long arms of chromosomes 17 [7] and 13 [8], respectively. and both apparently function as tumor suppressor genes. BRCA1 is a large protein of 1863 amino acids and BRCA2, with 3418 amino acids, is even larger. Both the proteins are involved in control of homologous recombination (HR) and double-strand break repair in response to DNA damage [9-14].

Mutations in the *BRCA1 *and *BRCA2 *genes were first reported in conjunction with their identification in 1994 [15] and 1995 [16,17]. To date 1536 distinct mutations, polymorphisms and variants in *BRCA1 *and 1885 in *BRCA2 *have been reported [BIC database, [18]], which are distributed throughout the entire coding regions of both genes. Together, mutations in both the genes account for the great majority of families with hereditary susceptibility to breast and ovarian cancer [19].

Epidemiological studies indicate that *BRCA1 *mutation carriers have a lifetime risk of breast cancer that is on the order of 60–80% [19-21]. The lifetime breast cancer risk for *BRCA2 *mutation carriers approaches that of *BRCA1 *carriers: however, disease onset has been documented to be at a later age [19,21]. In other words, women with an altered *BRCA1 *or *BRCA2 *gene are 3 to 7 times more likely to develop breast cancer than women without alterations in those genes [22], with very high relative risks for early disease onset (before age 40) of about 30-fold. Carriers of *BRCA1 *and *BRCA2 *mutation(s) are also at increased risk for other cancers – in particular, both genes increase the risk of ovarian cancer, while *BRCA2 *confers greatly increased risks of male breast cancer. Additional, but more modest risks are found for uterine, cervical, early-onset prostate and pancreatic cancers for *BRCA1 *[23], and prostatic, pancreatic, gallbladder, bile duct, stomach cancers and melanoma for *BRCA2 *[24].

The spectrum of *BRCA1 *and *BRCA2 *mutations has been characterized in different populations worldwide, with significant variation of the relative contribution of these genes to hereditary cancer between populations and examples of population specific founder mutations (*BRCA1*: 185delAG, 5382insC, *BRCA2*:6174delT in Jews, *BRCA2*: 999del5 in the Icelandic population) [reviewed in [25]]. However, the contribution of mutations in these two genes to breast cancer patients in the Indian population remains relatively unexplored apart from a few small studies [26-30]. Hence there is a need for screening a larger number of samples to investigate the role of *BRCA1/BRCA2 *gene mutations in the high-risk group of familial as well as early onset cases, which forms the largest group of breast cancer patients in the Indian population.

We have screened 204 breast cancer cases from North India for mutations throughout the entire coding region of both genes. The main focus behind the study is to provide reliable hospital based estimates of genetic influence, and to characterize the nature and prevalence of *BRCA1 *and *BRCA2 *germline mutations in early-onset and familial breast cancer cases.

## Methods

### Case selection

Two hundred and four patients of breast cancer referred from the department(s) of Surgery and Cancer Surgery Safdarjung Hospital, New Delhi and LLRM Medical College Meerut during 1999–2003 were selected for the study.

Selection of patients was mainly based on the following criteria: any patient with breast cancer diagnosed under the age of 40 years; any patient having a family history of breast or ovarian cancer; any patient having a previous personal history of ovarian cancer; and any male patient with or without family history. However, a few patients unslected for age of onset or family history were also included.

The study group included total **204 **patients (Table 1); 105(51.4%) early onset, 65(31.8%) late onset and 34 (16.6%) familial cases. Out of 204, 11 cases are bilateral (5 synchronous, 6 metachronous) and 8 are male breast cancer cases.

**Table 1:** Characteristics of Breast carcinoma patients.

Age group of patients	Total number of patients	Familial cases	Mutation positive cases (known deleterious mutations only)				
			
			*BRCA1*		*BRCA2*		**Total**
				
			F.H.	no F.H.	F.H.	no F.H.	
≤ 40	121	17 (14%)	0	2 (1.9%)	0	2 (1.9%)	4 (3.3%)
>40	83	17 (20.5%)	1 (5.9%)	0	0	1 (1.5%)	2 (2.4%)
**Total**	204	34 (16.7%)	1	2	0	3	6 (2.9%)

F.H. – family history; no F.H. – without reported family history.

The patient's ages ranged from 13 to 78 years with a mean age of 40.9 years (median 40 years). Informed consent was obtained from all participating patients and the study was carried out with the approval of Ethical Review Committee of Safdarjung Hospital, New Delhi.

To examine the population frequency of any sequence variants identified in the patients, a series of **140 **age-matched control samples were also collected from women attending antenatal checkups and blood bank donors in Delhi. The majority of control individuals were under 45 years (77%) and 71% were females.

### Blood sample collection

Peripheral blood samples (ca.10 ml) were collected into EDTA vials. The buffy coat was separated and frozen at -70°C for further use.

Genomic DNA was extracted from peripheral blood lymphocytes using a standard phenol-chloroform extraction method. Blood was first digested with lyses buffer I (30 mM Tris, 5 mM EDTA and 50 mM NaCl) and lyses buffer II (20% SDS, 100 μg/ml Prot.K) followed by the extraction with Tris saturated phenol and Chloroform-isoamyl alcohol (24:1) and finally recovered by ethanol precipitation.

### Mutation detection

The complete coding regions and exon-intron boundaries for both *BRCA1 *and *BRCA2 *genes were screened for DNA sequence variants by Heteroduplex analysis (HDX) of PCR amplicons using exon specific primers [31].

PCR reactions were carried out in a volume of 15 μl with 70–100 ng genomic DNA, 1× PCR buffer (20 mM Tris-Hcl pH 8.4,50 mM KCl), 1.5 mM MgCl2, 5 mM dNTP mix, 10 μM of both forward and reverse primer, 0.2 U platinum Taq (Invitrogen) and 0.4 μCi [α-P33] dATP (BRIT, Department of Atomic Energy, India). An initial de-naturation of 94°C for 3 min was followed by 40 cycles of amplification (30 s/94°C, 30 s/primer specific annealing temperature, and 30 s/72°C) and final elongation of 3 min/74°C.

Samples were diluted 1:1 in formamide dye (98% formamide, 10 mM NaOH, 0.05% bromophenol blue and 0.05% xylene cyanol) and 5 μl of each was loaded onto a HDX gel (40 × 40 cm; containing 0.5× MDE, 0.6× TBE, 4% glycerol, 400 μl 10%APS, 40 μl TEMED) and run at 8–10 mA for 16–20 hrs in 0.6× TBE at room temperature.

Gels were dried under vacuum at 80°C for 2 hrs and exposed to film (KODAK BioMax-MR Amersham, USA) for 10–12 hrs with an intensifying screen.

To rule out the possibility of PCR fidelity artefacts, both PCR amplification and gel based heteroduplex analysis was done twice for samples that showed altered mobility on HDX gels.

PCR products showing an aberrant banding pattern were re-amplified for sequencing using the same primers as for HDX analysis. DNA samples were sequenced either manually (Sequenase PCR Product Sequencing Kit and [α-33P] dATP (Amersham Life science) according to manufacturer's instruction) and by Automated genetic analysis (ABI 310/3100, Applied Biosystems, CA) to determine the exact sequence alteration identified. Samples were sequenced in both the forward and reverse sense to corroborate the findings.

## Results and discussion

The incidence of breast cancer in India has been increasing in recent years and is likely to pose an ever-increasing health care burden as socio-economic changes bring about increased exposure to lifestyle risk factors. The earlier average age of onset among Indian women compared to Western populations and the increased likelihood of early-onset disease being attributable to genetic susceptibility suggests the existence of a strong genetic component in this population. Evaluation of age of onset and family history among breast cancer patients diagnosed in Safdarjung Hospital, New Delhi supports these observations. Mean age of onset for 569 women diagnosed with breast cancer during 1989–2003 was 47.8 years, with the most common age group consisting of women 45–54 years (31.8%). Approximately 22% of cases were diagnosed under the age of 40 years. Of the 226 cases for whom family history information was available, 47 (20.7%) reported at least one additional breast or ovarian cancer case in first or second-degree relatives [32]. To determine the contribution of *BRCA1 *and *BRCA2 *to breast cancer in women of North India, we screened for alterations in the coding sequences and intron-exon boundaries of both genes in **204 **breast cancer patients and **140 **age-matched controls. This represents the largest study to date of the BRCA genes in the Indian population.

### Sequence variants in BRCA 1 & 2 genes

In total, **18 **sequence variants were identified in the study group, including **3 **frame shift (FS), **1 **missense (MS), **1 **5'UTR alteration, **1 **silent substitution, and **3 **intronic variants in *BRCA1 *(Table 2) and **3 **FS, **1 **MS, **1 **silent and **4 **intronic variants in the *BRCA 2 *gene (Table 3).

**Table 2:** BRCA1/BRCA2 deleterious mutations in Indian Breast Cancer patients

**Exon**	**Gene**	**NT**	**Base Change**	**Codon**	**AA Change**	**Designation**	**Variation type**	**BIC entry**	**Case(s) n = 204**	A/S/R*	**F.H**.	**Control(s) n = 140**
2	BRCA1	185	delAG	23	Stop 39	185delAG	Frame-shift protein truncating	Reported	1 (0.49%)	40/F/H	NO	0
11d	BRCA1	4184	delTCAA	1355	Stop 1634	4184del4	Frame-shift protein truncating	Reported	1 (0.49%)	60/F/H	3 Sisters + Mother Br Ca	0
11d	BRCA1	3596	delGAAA	1159	Stop 1208	3596del4	Frame-shift protein truncating	Reported	1 (0.49%)	24/F/M	NO	0
11e	BRCA2	6376	Ins AA	2049	Stop 2051	6376InsAA	Frame-shift protein truncating	Novel	1 (0.49%)	30/F/H	NO	0
19	BRCA2	8576	Ins C	2783	Stop 2797	8576InsC	Frame-shift protein truncating	Novel	1 (0.49%)	35/F/H	NO	0
27B	BRCA2	9999	del A	3258	Stop 3275	9999delA	Frame-shift protein truncating	Novel	1 (0.49%)	50/F/H	NO	0

*A-age of diagnosis; S-sex; R-religion (H-Hindu; M-Muslim) Br Ca-Breast Cancer.

**Table 3:** BRCA1/BRCA2 sequence variants of unknown significance and known polymorphisms in Indian Breast Cancer patients

**Exon**	**Gene**	**NT**	**Base Change**	**Codon**	**AA Change**	**Designation**	**Variation type**	**BIC entry**	**Case(s) n = 204**	**A/S/R^a^**	**Family History**	**Control(s) n = 140**
1	BRCA1	22	C>G	5'UTR	-	22C>G	Transition UV	Novel	1 (0.49%)	35/F/H	NO	0
7	BRCA1	560	T>C	Non coding	-	IVS7+38 T>C	Transversion Intronic, UV	Novel	1 (0.49%)	30/F/H	NO	0
11a	BRCA1	790	delG	Non coding	-	IVS10-12delG	Deletion Intronic, UV	Reported	4 (1.96%)	35/F/H 35/F/H 35/F/H 31/F/H	NO NO NO NO	0
11d	BRCA1	3668	A>G	1183	Lys to Arg	K 1183 R	Polymorphism	Reported	16 (7.84%)	-	-	25 (20.8%)^b^
13	BRCA1	4476	T>C	Non coding	-	IVS13+2 T>C	Transversion Intronic, UV	Reported	1 (0.49%)	30/F/H	NO	0
16	BRCA1	4956	A>G	1613	Ser to Gly	S1613G	Polymorphism	Reported	1 (0.49%)	62/F/H	NO	2 (1.7%)^b^
16	BRCA1	5075	G>A	1652	Met to Ile	M1652I	Polymorphism	Reported	14 (6.86%)	-	-	10 (8.3%)^b^
17	BRCA1	5119 5154	A>G C>T	1667 1679	Lys to Arg Leu to Leu	K1667R 5154 C>T	Transversion, Missense UV Transversion Silent, UV	Novel Novel	1 (0.49%) 1 (0.49%)	35/F/H	Mother +Sister Br Ca	0 0
2	BRCA2	295	T>A	Non coding	-	IVS2+90 T>A	Transition Intronic, UV	Novel	1 (0.49%)	32/F/H	NO	0
7	BRCA2	859	A>T	Non coding	-	IVS 7+75A>T	Transition Intronic, UV	Novel	1 (0.49%)	32/F/H	NO	0
8	BRCA2	909	C>T	Non coding	-	IVS 8+56C>T	Transversion, Intronic, UV	Novel	1 (0.49%)	52/F/H	Grand-Mother Br Ca	0
10B	BRCA2	1593	A>G	455	Ser to Ser	1593A>G	Transversion Silent, UV	Reported	1 (0.49%)	48/F/H	NO	0
14	BRCA2	7470	A>G	2414	Ser to Ser	7470A>G	Polymorphism	Reported	24 (11.8%)	-	-	32 (26.7%)^b^
22	BRCA2	9079	G>A	2951	Ala to Thr	A2951T	Transition Missense	Reported	1 (0.49%)	40/F/H	NO	0
25	BRCA2	9729	insG	Non coding	-	IVS25+58InsG	Substitution Intronic, UV	Novel	3 (1.47%)	45/F/H 30/F/H 24/F/H	NO NO Sister Br Ca	0

^a^A-age of diagnosis; S-sex; R-religion (H-Hindu; M-Muslim).

^b^Carrier frequency calculated from 120 controls, UV – unclassified variant, Br Ca-Breast Cancer.

Additional sequence variants detected in this study included **4 **common polymorphisms (*BRCA1*: K1183R in exon 11, S1613G and M1652I in exon 16; *BRCA2*: 7470A>G in exon 14) previously identified in other populations and reported in the BIC. These polymorphisms were observed in similar frequencies for both patients and controls.

No sequence alterations were observed in male and bilateral breast cancer patients.

### Frame-shift mutations in BRCA1

Three previously reported deleterious frame-shift mutations resulting in a premature termination codon were identified in *BRCA1*: 185delAG in exon 2; 3596del4 and 4184del4 in exon 11). These mutations were not observed in the control group.

The *BRCA1 ***185delAG **mutation was identified in an early onset index case [age 35] without any family history. This mutation is common in Ashkenazi Jews, having attained a 1% carrier frequency within the population [33] since origin of the ancestral mutation [34]. Population studies have shown that the 185delAG mutation predates the separation of Sephardi and Ashkenazi Jewish populations and is probably 2000 years old [35].

In India, 185delAG has been reported in all populations studied (Table 4) [26-30]. This deleterious frame shift mutation was first reported in a family residing in a part of Trivandrum not far from the small towns with settlement of Jewish people [26]. It was later reported in two South Indian families from Kerala province [29] as well as in two sisters from Goa, where a multi-ethnic population exists, with a significant influence of Portuguese (potential introduction of the mutation through Sephardic Jews) [30]. Surprisingly, we have found 185delAG in a North Indian Hindu patient residing in New Delhi who claimed to have no Jewish ancestry. Similarly, Lakhotia *et al*., in their initial screening found the same mutation in four Indian breast cancer families [36]. In addition to the clearly established founder effect for 185delAG, this mutation has been shown to have arisen independently at least twice [37], thus it would be interesting to evaluate the origin and population genetics of these disease susceptibility alleles in the Indian population through haplotype analysis, given the diverse multilingual, multireligious, and multiethnic roots of our society.

**Table 4:** BRCA1/BRCA2 mutations and sequence variants reported in Indian populations.

Gene	^a^Exon	^a^Nucleotide change	^a^Amino acid change	^b^Mutation type	^c^Mutation effect	Reported in BIC	^d^Saxena (2002); n = 20	^d^Saxena (2006); n = 204	^d^Kumar (2002); n = 14	^d^Valarmathi (2002); n = 13	^d^Valarmathi (2004); n = 16	^d^Hedau (2005); n = 124
BRCA1	1	22 C>G		5'UTR	UV	**No**		SC (35)				
BRCA1	2	185 delAG	fs23 Stop 39	FS	PT	Yes		SC (40)	≥ 1 FDR br/ov		F01: br (40,35,34) F09: br (51,59,45,54,45,30) ov (51)	FH+
BRCA1	2	147 G>A	Glu 10 Lys	MS	UV	**No**					patient/obligate carrier	
BRCA1	2	186 G>A	Glu 23 Lys	MS	UV	**No**					patient/obligate carrier	
BRCA1	IVS-5	331+1 G>T		SS	UV	Yes	FH+(30) br(36) ov(46)					
BRCA1	7	465 G>A	Glu 116 Lys	MS	UV	**No**			≥1 FDR br/ov			
BRCA1	7	448 A>C	Lys 110 Thr	MS	UV	**No**						FH+(40)
BRCA1	7	459 T>C	Ser 114 Pro	MS	UV	**No**						FH+(35)
BRCA1	IVS-7	560+38 T>C		NC	UV	**No**		SC (30)				
BRCA1	IVS-7	561-34 C>T		NC	PM	Yes						co-occurrence B1:185delAG
BRCA1	IVS-10	790-12 delG		NC	UV	**No**		SC (35) SC (35) SC (35) SC (31)				
BRCA1	11	1027 delA	fs303 Stop313	FS	PT	**No**			≥1 FDR br/ov			
BRCA1	11	3596 del4	fs1159 Stop1159	FS	PT	Yes		SC (24)				
BRCA1	11	3667A>G	Lys1183Arg	MS	PM	Yes		8% patients; 21% controls			patients and controls	
BRCA1	11	3672 G>T	Glu 1185 Stop	NS	PT	**No**					F08: br (36,45)	
BRCA1	11	3679 G>T	Ser 1187 Ile	MS	UV	Yes					patient/obligate carrier	
BRCA1	11	3730 G>T 3740 G>C	Arg 1204 Ile Lys 1207 Asn	MS MS	UV UV	**No** **No**					F12: patient co-occurrence	
BRCA1	11	3769C>A	Ser 1217 Tyr	MS	UV	**No**					patient/obligate carrier	
BRCA1	11	3867 G>T	Glu 1250 Stop	NS	PT	Yes				F1: br (42,40,34)		
BRCA1	11	3797 C>G 3846 A>G	Phe1226Leu Arg1243Gly	MS MS	UV UV	**No** **No**					F09: patient co-occurrence	
BRCA1	11	4184del4	fs1355 Stop1364	FS	PT	Yes		FH+ (60) br: (M,3S)				
BRCA1	12	4302 C>T	Gln 1395 Stop	NS	PT	Yes						FH+(40)
BRCA1	IVS-13	4476+2T>C		SS	UV	**No**	SC(30)	SC (30)				
BRCA1	16	4956 A>G	Ser 1613 Gly	MS	PM	Yes		0.5% patients; 1.7% controls				
BRCA1	16	5075 G>A	Met 1652 Ile	MS	UV	Yes		6.9% patients; 8.3% controls				
BRCA1	16	4956 insG	fs1613 Stop1621	FS	PT	**No**						FH+(45)
BRCA1	17	5119 A>G 5154 C>T	Lys 1667 Arg Leu 1679 Leu	MS silent	UV UV	**No**		FH+ (35) br: (M, S) co-occurrence				
BRCA1	IVS-18	5271+66A>G		NC	PM	Yes						co-occurrence B1:185delAG
BRCA1	20	5341 T>G	Val 1741 Gly	MS	UV	**No**				2.7% controls		
BRCA1	20	5364 C>G	Pro 1749 Ala	MS	UV	**No**				FH+ (30) FH+ (38)		
BRCA1	20	5379 G>T	Glu 1754 Stop	NS	PT	**Yes**				F2: br(40,39,32,29,27)		
BRCA2	2	203 G>A		5'UTR	PM	**Yes**					patients and controls	patients and controls
BRCA2	IVS-2	295+90 T>A		NC	UV	**No**		SC (32)				
BRCA2	IVS-3	545-54C>G		NC	PM	**No**					patients and controls	
BRCA2	IVS-7	859+75A>T		NC	UV	**No**		SC (32)				
BRCA2	IVS-8	909+56C>T		NC	UV	**Yes**		FH+ (52) br: (GM)				
BRCA2	10	1593A>G	Ser 455 Ser	silent	PM	**Yes**		SC (48)				
BRCA2	11	5227dupT 5639T>C 5929G>A	fs1667 Stop1676 Val1804Ala Glu1901Lys	FS MS MS	PT UV UV	**No** **No** **No**					F11: br (24) co-occurrence	
BRCA2	11	5242dupT		FS	PT	**No**					F03: br (45,41,28) ov (63)	
BRCA2	11	6180dupA	fs1984 Stop2002	FS	PT	**No**					F02: br (39,40,32,29,27)	
BRCA2	11	5624C>T 6515C>T	Thr 1679 Ile Pro 2096 Leu	MS MS	UV UV	**No** **No**					F12: br (36,45) co-occurrence	
BRCA2	11	5007A>C	Glu 1593 Asp	MS	UV	**Yes**	MBC(45) SC(32) not in cotrols					
BRCA2	11	6376 ins AA	fs2049 Stop2051	FS	PT	**No**		SC (30)				
BRCA2	14	7470A>G (PM)	Ser 2414 Ser	silent	PM	**Yes**		11.8% patients; 26.7% controls				
BRCA2	18	8345A>G	Asn 2706 Ser	MS	UV	**Yes**	SC(30) B1:IVS13 co-occurrence FH+(60) br(35,40) not in controls	-				
BRCA2	19	8576 insC	fs2783 Stop2797	FS	PT	**No**		SC (35)				
BRCA2	22	9079 G>A	Ala 2951 Thr	MS	PM	**Yes**		SC (40)				
BRCA2	IVS-25	9729+58InsGG		NC	UV	**No**		FH+ (24) br: S SC (30) SC (45)				
BRCA2	27B	9999delA	fs3258 Stop3275	FS	PT	**No**		SC (50)				

^a^Genbank BRCA1-HSU14680; Genbank BRCA2-; IVS – intervening sequence-intron number;

^b^UTR – untranslated region; NC – non-coding; FS – frame shift; MS – missense; NS – nonsense.

^c^SS – splice site; PT – protein truncating; MS – missense; UV – unclassified variant; PM – polymorphism.

^d^n is the total number of independent families studied; (age of dx.); FH+ – family history present (M-mother, S-sister; GM-grandmother); FDR – first degree relative; MBC-male breast cancer; SC – sporadic cases; br – breast cancer; ov – ovarian cancer; br/ov – breast, breast-ovarian or ovarian cancer

*BRCA1 ***3596del4 **was detected in a Muslim index case of very early onset disease [age 24] without any family history. Interestingly, the same mutation was reported in a heterogeneous Italian population intermixed with French, German, and Slovenian ethnic groups and a large number of Muslim immigrants [38]. These observations suggest that 3596del4 might be a common mutation in the Muslim community and might have migrated to the Indian population through a pool of Muslim immigrants.

The **4184del4 **mutation is located towards the C terminus of *BRCA1*, within the transcriptional activation domain [39,40], a region also reported to interact with the *BRCA2 *protein, which plays an important role in double stranded break (DSB) repair.

The above mutation was detected in a late onset index case [age 60] with a strong family history in first-degree relatives, thus belonging to a high-risk group. Her mother and three sisters were all affected with breast cancer. Interestingly, the same mutation was identified in the Pakistani population. One patient identified with this mutation was of Punjabi descent, and the other, who resided in Karachi, identified herself as Sindhi [41]. This mutation has been reported 107 times in the Breast Cancer Information Core (BIC) website in breast cancer cases of diverse ethnic origins [18]. In a study from northwest England, it was found that the 4184del4 mutation has at least three distinct haplotypic backgrounds, implying that the mutation has occurred independently on at least three occasions, suggesting this to be a mutational hotspot giving rise to recurrent mutations [42]. Haplotypic analysis would be interesting in this case also to determine whether 4184del4 has an independent origin in the Indian populaion or whether an existing mutation has been introduced through population admixture.

### Frame-shift mutations in BRCA2

Three protein truncating frame-shift mutations unique to Indian women (6376insAA, 8576insC, 9999delA) were observed in the *BRCA2 *gene.

The *BRCA2 *frame shift mutation **6376insAA **was found in an index case [age 30] without any family history. This mutation is located in the BRC repeats encoded in exon 11. These eight 30–40 residue motifs are conserved between several mammalian species, and have been shown to mediate binding of BRCA2 to the RAD51 protein, a mammalian protein essential for DNA repair and genetic recombination [43].

*BRCA2 ***8576insC **in exon 19 was detected in an early onset index case [age 35] without any family history.

The third mutation, **9999delA **in exon 27B, was found in a late onset case [age 50], also without any family history.

### Missense mutations in the BRCA1/2 genes

Apart from common polymorphisms, only a single missense mutation each was detected in *BRCA1 *and *BRCA2*. Neither mutation was detected in any control samples (280 chromosomes).

A novel *BRCA1 *missense, K1667R, along with a silent alteration 1679L in exon 17 was observed in the same high-risk index case [age 35] with a strong family history in first-degree relatives, thus suggesting its disease association.

One *BRCA2 *missense alteration A2951T was observed in exon 22. This mutation was observed in only one index case [age 40] without any family history. Originally reported as a polymorphism with a modest carrier frequency of 0.006 in an American population [44], our failure to detect this variant in any control samples suggests that this alteration may not represent a common polymorphism in the North Indian population.

### Functional significance

Missense mutations may be pathogenic, depending upon the nature of the amino acid substitution and its effect on protein structure or function. In general, missense alterations in conserved protein motifs are more likely to be deleterious. Missense amino acid changes in the p53-binding domain or the transactivation domain of *BRCA1 *adjacent to a BRCT repeat have been shown to be pathogenic [45,46]. Interestingly, the *BRCA1 *missense alteration K1667R is located in the BRCT domain. These conserved motifs are found in many other proteins, and are involved in DNA repair and cell cycle regulation. Tumor associated mutations are predicted to disrupt the folding or stability of the BRCT domain and thus effect protein function [46].

Non-conservative amino acid substitutions may disrupt protein folding, and *BRCA2 *A2591T leads to substitution of the non-polar hydrophobic amino acid alanine by polar hydrophilic threonine. However, although A2951 is invariant in vertebrate species through puffer fish, suggesting strong evolutionary conservation due to functional constraints, the frequency of this variant in disease-free individuals is counter indicative, and additional data are required to evaluate potential disease-association of this allele.

In addition to missense mutations, many variants outside of the exonic amino acid coding sequences were identified. One intriguing *BRCA1 *alteration, 22 C>G, was observed in the 5'UTR region which contains a consensus sequence of 5'-CCAGCCAUG-3' involved in the initiation of protein synthesis. This variant was detected in one patient but was absent in the control group. It may be possible that this mutation interferes with normal translational initiation of *BRCA1*, giving rise to a hypomorphic allele that may confer some risk of breast cancer; however, the exact functional significance remains uncertain.

While the exact functional relevance of the intronic variants identified in our study is not known, they may lead to aberrant splicing, either through alteration of consensus splice sites or other splice enhancer sequences. Exon skipping, the most frequent outcome, is thought to result from failure of the mutant splice sites to define an exon. *BRCA1 *IVS13 (+2) T>C in exon 13 was identified in the present study as well as in another patient in the previous pilot study [27]. This variant is within the consensus splice donor sequence, and hence could lead to aberrant splicing. Evaluating the effect of this variant on *BRCA1 *mRNA processing could support its functional significance. However, this awaits resampling of the respective mutation carriers, as at present, tissue samples for these patients are available only in the form of paraffin embedded blocks.

### Summary

We observed **9 **distinct *BRCA1 *and **9 **distinct BRCA2 sequence variants; 4 of the 9 *BRCA1 *(44%) and 7 of the 9 *BRCA2 *mutations (78%) are unique to the Indian population and are distributed throughout the exons of *BRCA1 *and *BRCA2 *gene. Of these 18 mutations, six clearly deleterious sequence variants were detected in 2.94% of the tested patients. The prevalence of BRCA1/2 mutations in our Indian patient series appears to be low compared to other Asian countries [[41,47] and [48]] but is comparatively similar to that reported from Shanghai China [49]. In addition, similar mutation frequencies were observed in series of early onset breast cancer cases in Britain [50].

It is possible that of the further 13 variants of uncertain clinical significance identified in 15 additional patients, some may be disease causal. These variants can be further evaluated in order to classify them into high- or low-risk categories based on epidemiological observations including degree of family history and segregation of the variant with disease. Additional classifiers include amino acid conservation, severity of amino acid change and evidence from functional assays [51].

In this study, scanning for the presence of sequence variation was performed by analyzing PCR amplicons using gel based HDX. It is estimated that the mutation detection sensitivity of the heteroduplex method utilized is approximately 80% [52]. Although the sensitivity of this approach is lower than some other mutation analysis techniques (e.g dHPLC, direct sequencing), it is relatively inexpensive, reasonably high throughput, technically simple to perform, and has been applied successfully for numerous genetic disorders – including *BRCA1 *and *BRCA2 *screening [31]. To increase the efficacy of mutation detection, the screening for mutations in both the genes was repeated by HDX in all familial cases. Moreover no currently available technique can guarantee 100% detection of pathogenic mutations in the BRCA1 and BRCA2 genes. In particular, all PCR based methods are unable to detect large genomic rearrangements that occur frequently in both genes and can account for a significant proportion of *BRCA1/BRCA2 *mutations [53-59].

Nevertheless, the number of clearly disease-associated mutations identified in the studied North-Indian population is lower than observed elsewhere. However, a significant proportion of women who had breast cancer diagnosed at age ≤40 years without any family history were carriers (all variants: 14.2%, known deleterious mutations: 3.8%). On the other hand, the identified mutations account for a comparatively small proportion of the familial risk of breast cancer (all variants: 11.7%, known deleterious mutations: 2.9%). This suggests one of several possibilities with respect to genetic predisposition in the North Indian population. First, there may be a significant proportion of *BRCA1/2 *mutations that are large germ line rearrangements, which would not have been detected by the method of mutation screening employed. Second, it is possible that there are some unknown genes, which may contribute more significantly to familial breast carcinoma in this population than do *BRCA1 *and *BRCA2*.

The present study is in agreement with the findings from our pilot study done on a small independent group of 20 breast cancer patients where 3 out of 5 cases with mutations in *BRCA1/2 *had early onset disease [27]. Thus, it is reasonable to postulate that women with early-onset disease without family history are likely to have a disease associated alteration of the *BRCA1 *or *BRCA2 *gene. A similar viewpoint has been put forwarded by a study conducted on Iranian women where it concluded that early onset breast cancer with a limited family history or without family history [60] is sufficient to justify mutation screening. It is possible that the significant number of early onset patients without reported family history have *BRCA1/2 *germ line mutations that are paternally inherited. In many cultures, knowledge of family history of disease is better documented along matrilineal lines, particularly those diseases that are gender restricted.

A possible explanation for the earlier age of disease onset in *BRCA1/2 *mutation carriers could be high circulatory estrogen levels in younger women compared to elderly women. According to one hypothesis, the total number of ovulatory cycles and thus exposure to higher estrogen level is a significant factor contributing to the risk of breast cancer [61]. Estrogen exposure is hypothesized to increase the susceptibility to breast tissue to carcinogenesis through continued cell division and proliferation resulting from multiple ovulatory cycles, principally between menarche and first birth, thus allowing for a concomitant increase in the accumulation of random genetic errors [62,63]. Increased estrogenic exposures increase the rate of proliferation, hence magnifying the effect. Studies have shown that wild-type *BRCA1 *blocks estrogen receptor (ER) mediated transcriptional activation, thereby inhibiting estrogenic signalling [64]. These observations could account for the higher proportion of early-onset breast cancer in populations where exposures to risk factors that primarily influence post-menopausal breast cancer risks are, as yet, relatively low.

Extensive mutation screening of high-risk breast cancer primarily targeting early-onset cases should be undertaken in India with proper genetic counseling, since female carriers of mutations in these genes are also at a high risk for developing a second malignancy either in the breast or ovary. Personal risk information may help in taking preventive measures and also motivate a high-risk woman to adopt breast screening that may promote early detection and improve chances of surviving breast cancer.

## Competing interests

The author(s) declare that they have no competing interests.

## Authors' contributions

SS conceived the study, and participated in its design and coordination and data analysis

AC carried out the molecular genetic studies, participated in the sequence alignment and drafted the manuscript.

MK carried out the molecular genetic studies.

C.I.S wrote the project proposal and manuscript, standardizing the research techniques and assisted with data analysis

D.E.G imparted training in research techniques and helped in drafting manuscript

SK, DB, R.S.M, Chintamani – helped in collecting samples and clinical details.

A.K.A, V.K.S – helped in collecting samples and clinical details.

P.C.S – participated in result analysis.

G.L. – conceived the study

All authors read and approved the final manuscript.

## Pre-publication history

The pre-publication history for this paper can be accessed here:


